# The relationship between the timing of pregnancy discovery and prenatal attachment and distress: a case-control study

**DOI:** 10.1590/1806-9282.20241399

**Published:** 2025-03-31

**Authors:** Yasemin Sökmen, Şükran Başgöl

**Affiliations:** 1 Ondokuz Mayıs University, Faculty of Health Sciences, Department of Midwifery – Samsun, Turkey.

**Keywords:** Pregnancy, Attachment, Recognition, Psychological distress

## Abstract

**OBJECTIVE:**

This research was conducted to determine the relationship between the timing of pregnancy discovery and prenatal attachment and distress.

**METHODS:**

An analytical, case-control research design was used. The study was conducted between April 2023 and March 2024. The population of the study consisted of pregnant women who presented to a training and research hospital in the north of Turkey for antenatal follow-up, and the sample consisted of 152 women from this population (case group 76 and control group 76). Data were collected using a Pregnant Descriptive Information Form, the Prenatal Attachment Inventory, and the Prenatal Distress Scale-Revised Version. The chi-square test, Mann-Whitney U test, and Spearman correlation analysis were utilized to analyze the data.

**RESULTS:**

The prenatal attachment scores of participants who discovered their pregnancies late were significantly lower than the scores of those whose pregnancies were discovered early (p<0.05). The prenatal distress scores of participants whose pregnancies were discovered late were significantly higher than the scores of those with early discovery (p<0.05). While a statistically positive, low-level relationship was detected between the prenatal attachment and prenatal distress scores of pregnant women whose pregnancies were discovered early (p<0.05), there was no statistically significant relationship between the scores of those who discovered their pregnancies late (p>0.05).

**CONCLUSION:**

There was a difference between the timing of pregnancy discovery and prenatal attachment and prenatal distress.

## INTRODUCTION

Pregnancy is a critical process that profoundly affects women's lives. In this process, pregnancy discovery after the eighth gestational week is called late pregnancy discovery^1^. Early or late pregnancy discovery may affect the chances of having a healthy pregnancy and baby, as well as causing the patient to miss the legal curettage time^1-4^. In addition, the timing of pregnancy discovery is a critical issue in terms of maternal and fetal health. During pregnancy, it is necessary to make positive health-related changes such as receiving prenatal care, using folic acid, starting health-enhancing practices like nutrition and exercise, changing the drugs used or rearranging their dose, and quitting harmful substances like tobacco or alcohol^1,2,4^.

Prenatal attachment involves the mother's expression of love for and bonding with her baby during pregnancy^5^. A pregnant woman who achieves attachment believes that her baby has established a relationship with her, imagines her baby, and sees it as an individual. Therefore, it has been reported that the pregnant woman shows love, compassion, and attention to her baby, protects it, and is sensitive toward it^5-7^. When the literature is examined, it can be seen that prenatal attachment is affected by the pregnant woman's education level, employment status, presence of social support, history of anxiety or depression, marital satisfaction, desire for pregnancy, history of abortion, diagnosis of risky pregnancy or fetal anomaly, and the risk for premature birth^5,8-11^.

In addition to physiological, psychological, and social changes during pregnancy, the thought of responsibility for the care of the baby in the postpartum period causes prenatal distress^12,13^. Prenatal distress is defined as the fear and anxiety that arise as a result of the pregnant woman's concerns about birth and the baby, her body image during pregnancy, and changes in her relationships with her partner and environment^13^. Factors such as low socioeconomic level, education level, age, unplanned pregnancy, and socioeconomic conditions lead to prenatal distress^14-18^. The review of the literature on the timing of pregnancy discovery has indicated that there are no studies that have involved women with a history of abortion and curettage in the examination of the effect of the timing of pregnancy discovery on prenatal attachment and prenatal distress^1,3,4^. In this regard, the data obtained from this study might contribute to the provision of education by health professionals for society and the organization of antenatal care services. The aim of this study is to determine the relationship between the timing of pregnancy discovery and prenatal attachment and distress.

## METHODS

### Type of the study

The study was planned as an analytical, case-control research.

### The setting and the properties of the study

The study was conducted at a training and research hospital in the north of Turkey between April 2023 and March 2024.

### Population and the sample of the study

The sample was determined using the G*Power 3.1.9.2 software. There was no study in the literature investigating the relationship between the timing of pregnancy discovery and prenatal attachment and distress. Therefore, it was decided to calculate the effect size of our study by taking the medium effect (0.50) in Cohen's effect size classification^19,20^. The sample size of the study was calculated as at least 128 pregnant women (case group 64, control group 64) according to the independent samples t-test results conducted using the G*Power 3.1.9.2 software, based on a confidence interval of 80% (1-α), a test power of 95% (1-β), and an effect size of d=0.50. To increase the analysis power of the study, a total of 152 pregnant women (case group 76 and control group 76) were included using the nonprobability sampling (random) method by increasing the sample size by 20%. During the data collection, a total of 47 pregnant women were excluded from the study as 17 were older than 35 years, 2 were diagnosed with psychological disorders (bipolar disorder, schizophrenia), and 28 did not want to participate in the study.

### Inclusion and exclusion criteria

In the study, women whose pregnancy was discovered in or before the eighth gestational week were included in the case group. In addition, both case and control groups included pregnant women who were between the ages of 18 and 35 years, were between the 4th and 40th weeks of gestation, presented to the pregnancy clinic for routine checkups, and were at least a primary school graduate. In the study, women who got pregnant with assisted reproductive techniques were diagnosed with psychological disorders, got pregnant as a result of sexual harassment, rape, or incest, or were diagnosed with fetal anomalies or risky pregnancies were not included in either group.

### Data collection tools

The data were collected using a Pregnant Descriptive Information Form, the Prenatal Attachment Inventory, and the Prenatal Distress Scale-Revised Version.

Pregnant Descriptive Information Form: This form was developed by the researchers following a literature review^1,3,5^. It involved a total of 24 questions, 10 about the sociodemographic characteristics of pregnant women (such as age and education level) and 14 about obstetric characteristics (such as the number of pregnancies).

The Prenatal Attachment Inventory: This form was developed by Muller in 1990 to make sense of the thoughts and feelings experienced by women during pregnancy and determine their attachment levels in the prenatal period^21^. Its Turkish validity and reliability study was conducted by Yılmaz and Beji^20^. Cronbach's alpha reliability coefficient of the inventory was reported as 0.84^21,20^. The alpha reliability coefficient of the scale was found to be 0.90 in this study.

The Prenatal Distress Scale (PDS)-Revised Version: This scale was first developed by Yali and Lobel^22^ to evaluate the concerns and anxieties specific to the pregnancy period^22^. The Turkish validity and reliability study was conducted by Yüksel et al^23^. Cronbach's alpha reliability coefficient of the scale was reported as 0.85^23^. The alpha reliability coefficient of the scale was found to be 0.80 in the present study.

### Data analysis

The research data were analyzed using the Statistical Package for Social Science27. Before statistical comparisons between groups with early and late pregnancy discovery were made, their suitability for normal distribution was examined with the Kolmogorov-Smirnov test. Mann-Whitney U test was used to compare non-normally distributed scale scores based on questions with two response options. The chi-square independence test was preferred when testing the significance of relationships between categorical data. In cases where the relationships between numerical variables were not normally distributed, Spearman's correlation test was employed. The significance level was p<0.05.

### Ethical aspects of the study

This study was conducted in accordance with the principles of the Declaration of Helsinki. Ethics committee approval for the research was obtained from the Ondokuz Mayıs University Social and Human Sciences Ethics Committee (protocol no: 2022-865). The pregnant women's verbal and written consent was obtained.

## RESULTS

According to these results, there was no statistically significant relationship between the participants’ family type, employment status, and perceived income and the time when their pregnancies were discovered (p=0.100, 0.797, and 0.346, respectively). When we look at the distribution by age groups, while the majority of the 18–24-year-old and 25–30-year-old groups were diagnosed early, the pregnancies of 61.8% of the 31–35-year-old participants were discovered late. In addition, it was determined that the median age of the participants who discovered their pregnancy late (31) was higher than the median age of the participants who discovered it early (28), and this difference was statistically significant (p=0.001). In addition, it was determined that as participants’ education level increased, the rate of early pregnancy discovery also increased, and there was a statistically significant difference between the timing of pregnancy discovery and education level (p=0.002) (Table 1).

**Table 1:** Comparison of the timing of pregnancy discovery according to participants’ sociodemographic and obstetric characteristics.

Characteristics		n (%)	Timing of pregnancy discovery		Test statistics	p
			Early (n=76)	Late (n=76)		
			n (%)	n (%)		
Age						
	18–24	30 (19.8)	17 (56.7)	13 (43.3)		
	25–30	61 (40.1)	40 (65.6)	21 (34.4)		
	31–35	61 (40.1)	19 (31.1)	42 (68.9)		
	Median (min–max)	29 (19–42)	28 (19–40)	31 (20–42)	3.029	**0.001** U
Family type						
	Core	131 (86.2)	69 (52.7)	62 (47.3)	2.207	0.100^χ2^
	Extended	21 (13.8)	7 (33.3)	14 (66.7)		
Education						
Elementary/middle	49 (32.2)		15 (30.6)	34 (69.4)	12.070	**0.002** ^χ2^
	High school	62 (40.8)	34 (54.8)	28 (45.2)		
	Undergraduate	41 (27.0)	27 (65.9)	14 (34.1)		
Employment						
	Yes	17 (11.2)	9 (52.9)	8 (47.1)	0.066	0.797^χ2^
	No	135 (88.8)	67 (49.6)	68 (50.4)		
Place of residence						
	Village/town	10 (6.6)	3 (30.0)	7 (70.0)	–	–
	County	59 (38.8)	26 (44.1)	33 (55.9)		
	Provincial center	83 (54.6)	47 (56.6)	36 (43.4)		
Economic status						
	Income<expenses	31 (20.4)	17 (54.8)	14 (45.2)	2.121	0.346^χ2^
	Income=expenses	100 (65.8)	46 (46.0)	54 (54.0)		
	Income>expenses	21 (13.8)	13 (61.9)	8 (38.1)		
**Gestational week**						
	29–40	152 (100.0)	76 (100.0)	76 (100.0)	-0.293	0.770^U^
	Median (min–max)	37 (31–41)	37 (31–40)	37 (32–41)		
Number of pregnancies						
	1	42 (27.6)	29 (69.0)	13 (31.0)	8.243	
	≥2	110 (72.4)	47 (42.7)	63 (57.3)		
	Median (min–max)	2 (1–8)	2 (1–8)	2 (1–6)	3.730	**0.001** U
Number of live births						
	0	57 (37.5)	38 (66.7)	19 (33.3)		
	1	58 (38.2)	29 (50.0)	29 (50.0)		
	≥2	37 (24.3)	9 (24.3)	28 (75.7)		
	Median (min–max)	1 (0–3)	1 (0–2)	1 (0–3)	4.082	**<0.001** U
Status of having a curettage						
	Yes	20 (15.2)	9 (45.0)	11 (55.0)		
	No	132 (86.8)	67 (50.8)	65 (49.2)		
	Median (min–max)	0 (0–4)	0 (0–2)	0 (0–4)	0.531	0.343^U^
Status of having a miscarriage						
	Yes	21 (13.8)	9 (42.9)	12 (57.1)		
	No	131 (86.2)	67 (51.1)	64 (48.9)		
	Median (min–max)	0 (0–4)	0 (0–4)	0 (0–3)	0.682	0.595^U^
Planned pregnancy						
	Planned	88 (57.9)	60 (68.2)	28 (31.8)	27.636	**<0.001** ^χ2^
	Unplanned	64 (42.1)	16 (25.0)	48 (75.0)		
Number of antenatal follow-ups						
	<4	144 (94.7)	73 (50.7)	71 (49.3)	–	–
	≥4	8 (5.3)	3 (37.5)	5 (62.5)		

U Mann-Whitney U test; ^χ2^independent samples chi-square test. Bold indicates p<0.05.

In both groups, pregnancy was discovered early in 69% of the first pregnancies, but it was discovered late in 57.3% of multiparous participants, and there was a statistically significant difference between the two groups (p=0.001). It was determined that the number of live births in participants who discovered their pregnancy late was significantly higher than those in participants who did it early, and there was a statistically significant difference between the groups (p=0.001). In addition, 68.2% of the participants who had a planned pregnancy discovered their pregnancy early, while 75% of those who had an unplanned pregnancy discovered their pregnancy late, and there was a statistically significant difference between the two groups in terms of planned pregnancy (p=0.001) (Table 1).

According to these results, the prenatal attachment scores of participants whose pregnancy was discovered late (55) were lower than the scores of those whose pregnancy was detected early (72), and this difference was statistically significant (p=0.001). The prenatal distress scores of participants who discovered pregnancy late (13) were higher than those who did it early (9), and this difference was statistically significant (p=0.001) (Table 2).

**Table 2:** Comparison of participants’ mean prenatal attachment and prenatal distress scores according to the timing of pregnancy discovery.

Scales	Total		Timing of pregnancy discovery				Test statistics/p
				Early (n=76)	**Late (n=76)**		
	Mean±SD*	Median (min–max)	Mean±SD*	Median (min–max)	Mean±SD*	Median (min–max)	
PAI	63.37±11.83	68 (29–100)	72.36±5.41	72 (53–100)	54.38±9.44	55 (29–74)	-9.901 **<0.001** U
PDS-R	11.82±6.27	11 (2–32)	9.81±4.48	9 (2–21)	13.84±7.13	13 (3–32)	3.383 **0.001** U

* Mean±standard deviation;

U Mann-Whitney U test; PAI: Prenatal Attachment Inventory; PDS-R: Prenatal Distress Scale-Revised Version; SD: standard deviation. Bold indicates p<0.05.

**Figure 1:**
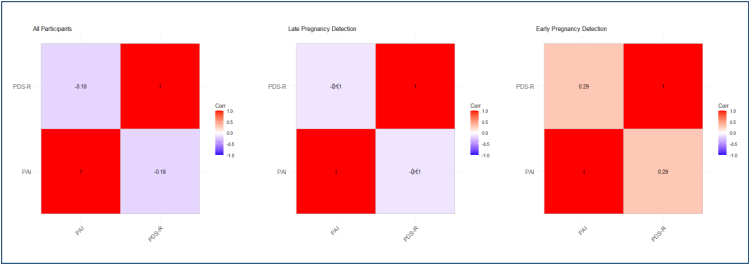
Relationships between participants’ prenatal attachment and prenatal distress scores

When all the participants were examined, a statistically low, negative relationship was found between prenatal attachment and prenatal distress scores (p=0.03). According to the findings of the participants whose pregnancies were discovered early, a low-level, statistically positive relationship was found between prenatal attachment and prenatal distress scores (p=0.011). However, no statistically significant relationship was discovered between prenatal attachment and prenatal distress scores of participants whose pregnancy was discovered late (p=0.334), (Table 3); (Figure 1).

**Table 3:** Relationships between participants’ prenatal attachment and prenatal distress scores.

Group	Scale	Value	PAI	PDS-R
Total	PAI	s	1.000	
		p		
	PDS-R	s	-0.177	1.000
		p	**0.030**	
Early discovery	PAI	s	1.000	
		p		
	PDS-R	s	0.289	1.000
		p	**0.011**	
Late discovery	PAI	s	1.000	
		p		
	PDS-R	s	-0.112	1.000
		p	0.334	

S: Spearman's correlation test; p: significance level; PAI: Prenatal Attachment Inventory; PDS-R: Prenatal Distress Scale-Revised Version. Bold indicates p<0.05.

## DISCUSSION

In this study, which was conducted to determine the relationship between the timing of pregnancy discovery and prenatal attachment and distress, it was found that the mean prenatal attachment scores of participants whose pregnancy was discovered late were lower than the scores of those whose pregnancy was detected early, while their mean prenatal distress scores were higher, and that this difference was significant. There were no data about prenatal attachment and distress in studies on the timing of pregnancy discovery in the literature; therefore, no comparison could be made^1-4^. It is thought that early pregnancy discovery affects the transition and adaptation process to parenthood, reducing the pregnant woman's prenatal distress level and, in parallel, increasing the prenatal attachment level. On the other hand, late pregnancy discovery increases the level of prenatal distress because it causes delays in positive health behaviors such as quitting smoking and starting folic acid supplements. This study presents new findings, which have not been evaluated before, to the literature. Additionally, a statistically negative relationship was found between pregnant women's prenatal attachment and prenatal distress scores. Similar to our research findings, studies in the literature have shown that as the prenatal distress level of pregnant women increases, their prenatal attachment level decreases^17,18^.

Many factors related to the timing of pregnancy discovery in different cultures and societies affect maternal and child health and therefore public health outcomes^2,4^. In this study, while there was no significant difference between pregnant women's timing of pregnancy discovery and their family type, employment status, place of residence, and perceived economic status, a significant difference was found in terms of their age, education levels, and pregnancy planning status. Similar to our research finding, in the study in which the National Survey of Family Growth data were analyzed, it was stated that there was a relationship between late pregnancy discovery and age, perceived economic status, education level, and unwanted or unplanned pregnancy^1^. In addition, some other studies have shown that there is a relationship between late pregnancy discovery and pregnant women's age, income level, marriage status, ethnic group, and marital adjustment^24^.

### Strengths and limitations

Since the participants were recruited from only one hospital, the results cannot be generalized across the country; however, since the hospital where the data were collected was one of the largest hospitals in the province, our results can be generalized throughout the province as many pregnant women from surrounding provinces and districts present to the gynecology clinic of this institution. Additionally, no generalization can be made across the country due to regional and cultural differences. The strength of the study is that the new findings obtained as a result of the study will contribute to the literature and provide guidance for future research.

## CONCLUSION

This study found that the mean prenatal attachment scores of participants whose pregnancy was detected late were lower than the scores of those whose pregnancy was detected early, while their mean prenatal distress scores were higher. Health professionals, especially midwives and nurses, who provide primary health care, need to recognize the factors affecting the discovery of pregnancy and teach all women of reproductive age the signs and symptoms of pregnancy.

## ETHICAL CONSIDERATIONS

This study was performed in line with the principles of the Declaration of Helsinki. Approval was granted by Ondokuz Mayıs University Social and Human Sciences Research Ethics Committee (October 28, 2022/no. 2022-865).

## CONSENT

Informed consent was obtained from all individual participants included in the study.

## CONSENT TO PUBLISH

The authors affirm that human research participants provided informed consent for the publication of the image in Figure 1a.
